# +874(T→A) single nucleotide gene polymorphism does not represent a risk factor for Alzheimer's disease

**DOI:** 10.1186/1742-4933-1-6

**Published:** 2004-11-12

**Authors:** Lorenza Galimberti, Beatrice Arosio, Carmen Calabresi, Silvia Scurati, Susanna Hamilton, Simona Delli Carpini, Carlo Vergani, Giorgio Annoni

**Affiliations:** 1 Department of Geriatrics, Ospedale Maggiore IRCCS, University of Milano, Milan, Italy; 2 Department of Clinical Medicine, Prevention and Medical Biotechnology, University of Milano-Bicocca, Mila, Italy

## Abstract

In the recent years, several cytokines have been associated with Alzheimer's disease (AD) development and progression and many studies have correlated this risk with polymorphisms in the genes encoding these molecules. Also the type 1 cytokine interferon (IFN)-γ belongs to a cytokine class that affects the immune function; in fact it plays a major role in defence against viruses and intracellular pathogens but also in the induction of the immune-mediated inflammatory response. The aim of this study was to evaluate the role of IFN-γ in AD by studying the association of +874T→A IFN-γ gene polymorphism with AD. We included in this study 115 AD patients (70 women, 45 men, mean age 80) and 90 sex and age-matched healthy controls (HC, 51 women, 39 men, mean age 82) from northern Italy. Genomic DNA was extracted with the salting-out method from whole blood of all subjects; the genotyping at IFN-γ loci was assessed with ARMS-PCR. The data obtained from the +874T→A IFN-γ gene polymorphism analysis of AD patients and HC lack of any statistically significant differences also when stratified according to gender. In conclusion these results confirm the previous shown lack of association between +874T→A IFN-γ gene polymorphism and the risk of AD. However, other polymorphisms have been demonstrated to influence IFN-γ transcription and since natural killer cells of AD patients show higher production of the cytokine, further analysis will be necessary to clarify the role of this gene in the pathogenesis of the disease.

In the human brain several cell types are responsible for initiating and amplifying a specific inflammatory response. In Alzheimer disease (AD) signs of an inflammatory activation of microglia and astroglia are present both inside and outside amyloid deposits. Cell cultures and animal models suggest an interactive relationship between inflammatory response activation, reduced neuronal functioning and amyloid deposition. Furthermore cells associated with extracellular plaques within AD brains can produce a variety of cytokines, chemokines and other related proteins that influence plaque and tangle formation [1]. For these reasons cytokines could play a critical role in the pathogenesis of AD. In the recent years, several cytokines have been associated with AD development and progression and many studies have correlated this risk with polymorphisms in the genes encoding these molecules [2-4]. Inside this research area we described that single nucleotide polymorphisms (SNP)s of the intereleukin(IL)-10 and IL-6 genes were associated with highest risk of AD with apparent interaction between these two genes [5]; these results supported the theory that the overall risk of developing AD may be governed by a 'susceptibility profile' and reflected the combined influence of inheriting multiple high-risk alleles. Also the type 1 cytokine interferon (IFN)-γ belongs to a cytokine class that affects the immune function; in fact it plays a major role in defence against viruses and intracellular pathogens but also in the induction of the immune-mediated inflammatory response [6]. It has been reported that the polymorphism +874(T→A) of the gene encoding IFN-γ is associated with a different production of this molecule, in particular the T allele correlates with increased levels of the cytokine [7]. In regards to the hypothesis that IFN-γ SNP may represent a genetic risk factor for AD, a recent study did not support this possibility [8]. Therefore with reference to this paper we try to confirm their hypothesis in a sample of patients, that were already genotyped for apolipoprotein E (ApoE), IL-10 and IL-6 [5]. We included in this study 115 AD patients (70 women, 45 men, mean age 80 ± 2) and 90 sex and age-matched healthy controls (HC, 51 women, 39 men, mean age 82 ± 2) from northern Italy. The clinical diagnosis of AD fulfilled the international criteria of the DMS IV and NINCDS-ADRDA; every patient had a recent brain magnetic resonance imaging (MRI)/computed tomography (CT) scan available. Cognitive performances were assessed according to the Mini-Mental State Evaluation (MMSE). Only AD and HC without clinical signs of inflammation (e.g. normal body temperature, no concomitant inflammatory condition) were eligible in order to minimize the risk of clinical or sub-clinical inflammatory processes. Blood chemistry tests were done and subjects with an abnormal red blood cell sedimentation rate or altered albumin and transferrin plasma levels were excluded. AD patients were further selected according to their C reactive protein (CRP) plasma levels and anyone with CRP above 5 mg/L (mean + 2 standard deviations of control values) were not eligible. Informed consent was obtained from all the subjects or their relatives. Genomic DNA was extracted with the salting-out method from whole blood of all subjects; the genotyping at IFN-γ loci was assessed with the same amplification method (ARMS-PCR) described by Scola et al. [8]. The genotype frequencies in the study groups were compared by the chi-square (χ) test in order to calculate significant different SNP distribution between AD patients and controls (Table 1). The data obtained from the +874T→A IFN-γ gene polymorphism analysis of AD patients and HC lack of any statistically significant differences also when stratified according to gender (data not shown). The percentage of the different genotypes was similar in AD compared with HC (T/T: 17.4% vs. 16.8% ; T/A: 50.4% vs. 55.1% ; A/A 32.2% vs. 28.1%) and consequently also the allele distribution shows no differences (T: 42.6% vs. 44.4% ; A: 57.4% vs. 55.6%). Our genotype distribution looks very similar to that found by Scola et al. [8]. In conclusion these results confirm the lake of association between +874T→A IFN-γ gene polymorphism and the risk of AD. Nevertheless other polymorphisms have been demonstrated to influence IFN-γ transcription and since natural killer cells of AD patients show higher production of the cytokine, further analysis will be necessary to clarify the role of this gene in the pathogenesis of the disease [9,10].

**Table 1:** IFN-γ genotype and allele distribution

	**Genotype**			**Allele**	
	**T/T^(H)^**	**T/A^(I)^**	**A/A^(L)^**	**T**	**A**

**AD**	20(17,4%)	58(50,4%)	37(32,2%)	98(42,6%)	132(57,4%)
**HC**	15(16,8%)	49(55,1%)	25(28,1%)	79(44,4%)	99(55,6%)

Genotype χ^2 ^= 0.488, df = 2, p = 0.783

Allele χ^2 ^= 0.232, df = 1, p = 0.63

In brackets there are the corresponding phenotype high (H), intermediate (I) and low (L).
